# Hypertrophic cardiomyopathy patients have a steep left ventricle to aortic root angle compared to normal as demonstrated on 3-D Tomographic Imaging: a case-control study

**DOI:** 10.1186/1532-429X-11-S1-P143

**Published:** 2009-01-28

**Authors:** Deborah H Kwon, Nicholas G Smedira, Zoran B Popovic, Bruce W Lytle, Randolph M Setser, Maran Thamilarasan, Paul Schoenhagen, Scott D Flamm, Harry Lever, Milind Y Desai

**Affiliations:** Cleveland Clinic Foundation, 9500 Euclid Avenue, Cleveland, OH 44195 USA

**Keywords:** Body Surface Area, Cardiac Magnetic Resonance, Hypertrophic Cardiomyopathy, Left Ventricular Outflow Tract, Aortic Disease

## Introduction

**Figure 1:**
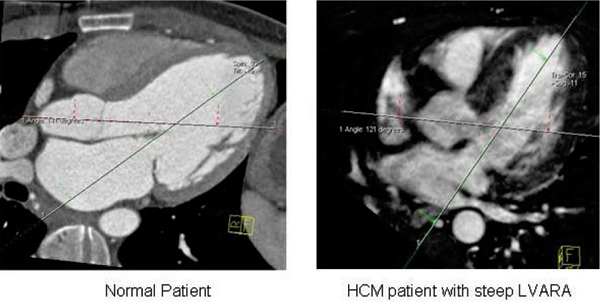
**Quantification of LVARA using the 5 chamber view**.

Hypertrophic cardiomyopathy (HCM) is characterized by disproportionate left ventricular (LV) hypertrophy, which cannot be attributed to other concomitant cardiac or systemic diseases. HCM can result in accelerated cardiac remodelling; thus affecting the pathophysiology of the disease. Because of an increased utilization of cardiac magnetic resonance (CMR) in the diagnosis and management of HCM, we have observed that a subgroup of HCM patients had a steep LV to aortic root angle (LVARA, Figure 1).

## Purpose

We conducted a case-control study to assess if HCM patients have a steeper LVARA as compared to normal subjects. Furthermore, we sought to discern the potential predictors of a steeper LVARA.

## Methods

We studied 153 consecutive patients (≤ 65 years) with echo-documented HCM that underwent standard CMR (1.5 T Siemens Avanto, Erlangen, Germany) along with whole-heart 3D MR angiogram which was a navigator-assisted free-breathing, ECG-triggered, fat saturated, T2-prepared, segmented 3D SSFP sequence. Imaging parameters were as follows: TR = 3.8 ms, TE = 1.9 ms, flip angle = 70°, acquired matrix = 175–216 (phase direction) by 256 (readout direction) points (no interpolation), Grappa factor of 2, 24 reference lines and sampling bandwidth = ± 125 kHz. Typically, 60–70 slices were acquired at 1.5 mm thickness (interpolated) in order to cover the heart. The in-plane resolution was typically 1.3–1.6 mm. Images were acquired during a 150 msec data acquisition window in mid-to-late diastole. LVARA, LV volumes (indexed to body surface area) and basal septal thickness (BST) were measured on CMR. We also studied 42 controls (< 65 years of age, < 1.2 cm diastolic BST, without hypertension, valvular, myocardial or aortic diseases) that underwent contrast-enhanced 64-multi-detector computed tomography (MDCT) coronary angiography (90 cc iodinated contrast). No controls had coronary artery disease or LV dilatation.

## Results

The baseline characteristics of HCM patients were as follows: mean age 46 ± 14 years, 68% male, 36% hypertensives, and 73% were on beta-blockers. The mean age of the controls was 43 ± 11 years and 38% women. There was a significant difference in BST (1.98 cm ± 0.64 vs. 0.99 cm ± 0.14) and LVARA between HCM patients and controls (134° ± 10 vs. 140° ± 7, p = 0.001). There was a significant inverse correlation between LVARA and age in both, HCM (beta = -0.56, p < 0.001) and control (beta = -0.48, p < 0.001) groups. In the HCM group, the mean body surface area, end systolic and end diastolic volume indexes were 134° ± 1.98 cm ± 0.6, 32 ml/m^2^ ± 11 and 84 ml/m^2^ ± 16, respectively. Within the HCM group, the univariate and multivariate associations between LVARA and various potential predictors are shown in Table 1.

**Table 1:** Univariate and multivariate regression analysis testing the association between LV-aortic root angle

	Univariate Analysis Beta	Univariate Analysis p value	Multivariate Analysis Beta	Multivariate Analysis p value
Age	**-0.56**	**< 0. 0001**	**-0.61**	**< 0. 0001**
Basal septal thickness	**-0.18**	**0.02**	**-0.25**	**< 0.01**
Body surface area	**-0.20**	**0.02**	**-0.16**	**0.02**
Hypertension	-0.19	0.02	0.09	0.20
Coronary artery disease	-0.17	0.04	0.05	0.4
Betablockers	-0.16	0.05	0.002	0.98
Gender	-0.16	0.05		
End-systolic volume index	0.11	0.17		
End-diastolic volume index	-0.007	0.9		

## Conclusion

As compared to normal subjects, patients with HCM have a significantly steeper LVARA. The strongest predictor of a steep LVARA is age, followed by BST and body surface area. In HCM patients, a steep LVARA, which likely represents remodelling of the LV, might result in increased turbulence across the LV outflow tract. This finding may also contribute to the complex pathophysiology of dynamic LVOT obstruction. CMR can accurately discern the LV-aortic morphology and angulation.

